# Sustainable Stress Management: Aquatic Plants vs. Terrestrial Plants †

**DOI:** 10.3390/plants12112208

**Published:** 2023-06-03

**Authors:** K. Sowjanya Sree, Klaus J. Appenroth, Ralf Oelmüller

**Affiliations:** 1 Department of Environmental Science, Central University of Kerala, Periye 671320, India; 2 Matthias Schleiden Institute—Plant Physiology, Friedrich Schiller University of Jena, 07743 Jena, Germany

**Keywords:** abiotic stress management, biotic stress management, duckweed

## Abstract

The Indo-German Science and Technology Centre (IGSTC) funded an Indo-German Workshop on *Sustainable Stress Management: Aquatic plants* vs. *Terrestrial plants* (*IGW-SSMAT*) which was jointly organized at the Friedrich Schiller University of Jena, Germany from 25 to 27 July 2022 by Prof. Dr. Ralf Oelmüller, Friedrich Schiller University of Jena, Germany as the German coordinator and Dr. K. Sowjanya Sree, Central University of Kerala, India as the Indian Coordinator. The workshop constituted researchers working in this field from both India and Germany and brought together these experts in the field of sustainable stress management for scientific discussions, brainstorming and networking.

## 1. Introduction

Climate change and the rapid growth of the human population are major global challenges. Evolving sustainable strategies to counter their effects will be the tasks for the future. We need to produce more food, which includes higher crop yields per area, with plants that are better adapted to the upcoming threats, while nature and climate need to be protected simultaneously (cf. United Nations Sustainable Development Goals). Besides crop production for food and feed, the growing human population and modern technology require plant biomass also for energy production, in the chemical and pharmaceutical industries, in housing and in the textile industry. Further to the promotion of our knowledge on resistance mechanisms in utilized crop species, higher crop yields and biomass production require novel approaches such as the use of new non-conventional crop species and the exploration of new agriculturally suitable areas. Finally, the scientific achievements obtained with conventional crop plants must be tested and eventually transferred to newly introduced crop species, if possible, or new strategies need to be developed for new crop species and new agricultural systems.

**Figure 1:**
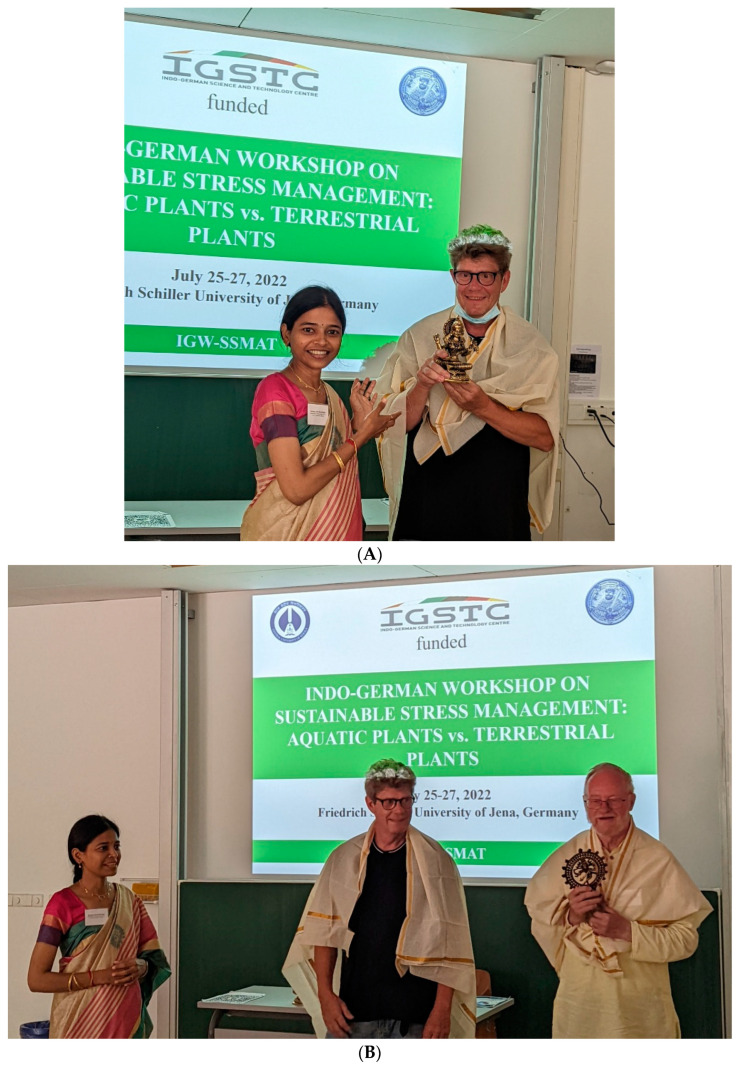
Inauguration of the Indo-German Workshop—SSMAT. (**A**) K. Sowjanya Sree (Indian coordinator) felicitating Ralf Oelmüller (German coordinator). (**B**) K. Sowjanya Sree felicitating Klaus-J. Appenroth (Right) in the presence of Ralf Oelmüller (Center).

**Figure 2:**
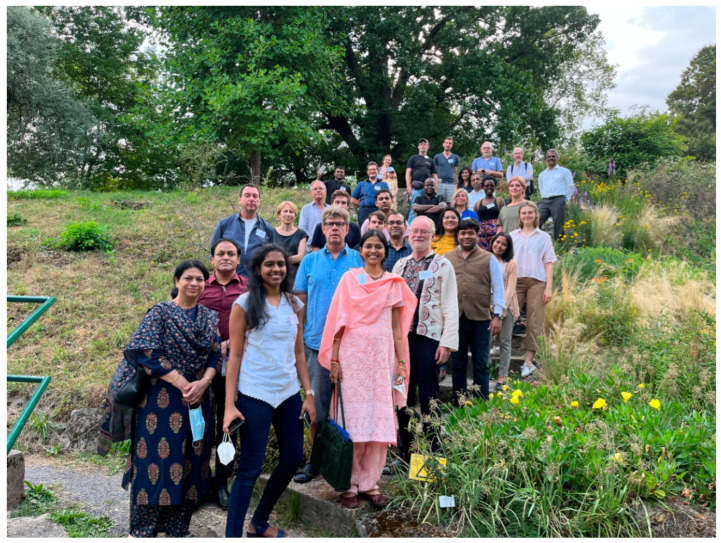
Group photo of the IGW-SSMAT participants.

**Figure 3:**
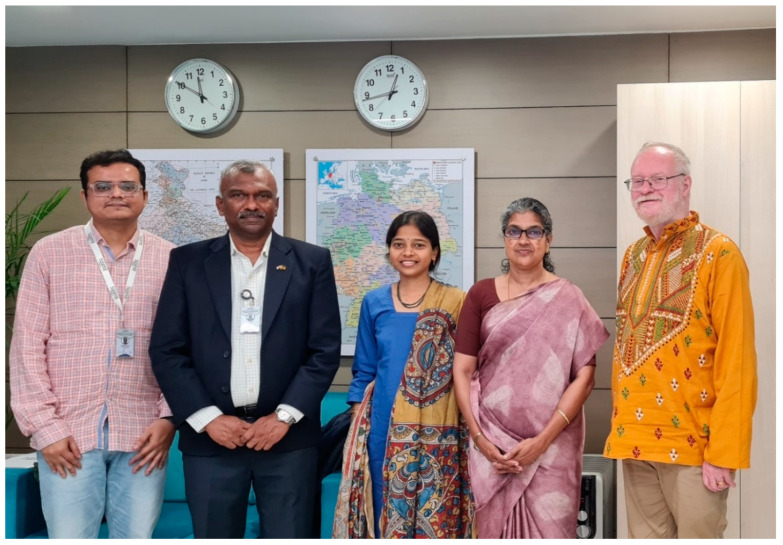
Scientific discussion between the IGW-SSMAT team and IGSTC team. From left to right: Saquib Shaikh, Deputy Scientific Officer, IGSTC; R. Madhan, Director, IGSTC; K. Sowjanya Sree, Central University of Kerala, India; P. V. Lalitha, Senior Scientific Officer, IGSTC; Klaus-J. Appenroth, University of Jena, Germany.

In this context, the Indo-German Workshop on *Sustainable Stress Management: Aquatic plants* vs. *Terrestrial plants* (*IGW-SSMAT*) was organized at the Friedrich Schiller University of Jena, Germany from 25 to 27 July 2022. This workshop was funded by the Indo-German Science and Technology Centre (IGSTC) with Prof. Dr. Ralf Oelmüller, Friedrich Schiller University of Jena, Germany, as the German coordinator and Chair of the workshop and Dr. K. Sowjanya Sree, Central University of Kerala, India, as the Indian Coordinator and Co-chair of the workshop. The scientific organization of the workshop was supported by Dr. Klaus J. Appenroth, Friedrich Schiller University of Jena, Germany. Interestingly, the workshop was held in a seminar room of the Department of Indo-German Studies, University of Jena, Germany, which was an apt location for the exchange of scientific ideas between the researchers of the two countries (Figure 1). The workshop included 25 oral presentations, 4 e-poster presentations, and a total of 38 invited and additional participants (Figure 2), which was an ideal group for one-to-one scientific discussions, group discussions, brainstorming sessions and for networking between the scientists from the two countries. The efforts of the IGSTC (Figure 3) in bringing together leading scientists in this field from both countries had synergistic effects on concept development, project coordination, grant applications, and contacts to industry.

The introduction of aquatic plants such as duckweeds into agricultural concepts for the future has opened novel research and application fields. Aquatic plants take up the nutrients from water for their growth and utilize space that otherwise is not used for agriculture. Therefore, comparative analyses of these crops with conventional land crops may contribute to a faster introduction of aquatic plants into agricultural applications. Besides knowledge transfer, novel strategies for large scale applications were also discussed. For instance, space limitation for the growth of land plants has promoted strategies which act on individuals (more biomass and better resistance), whereas strategies that allow the faster propagation of the plant population under different environmental conditions might be more important for aquatic plants.

Duckweeds (Lemnaceae) are on their way to becoming an important new crop plant. This is the result of more than a decade of intensive research, which revealed interesting properties of this family of aquatic plants [1]. Under optimal growth conditions, they contain high amounts of protein and an amino acid spectrum that fits very well to the nutritional requirements of humans, and also possess a high-quality fatty acid spectrum (although low in content), rich mineral composition, antioxidants and phytosterols [2,3,4]. Very recently, the European Union Commission permitted the sale of fresh duckweed plants (*Wolffia arrhiza* and *Wolffia globosa*) for human nutrition under the law of novel food [5]. Therefore, in the present, their use for human nutrition, and perhaps for the feeding of selected animals like pets, seems to be the most promising application of duckweeds. These qualitative advantages meet one important physiological property: duckweeds represent the fastest growing angiosperms [6,7]. Beside their possible use in nutrition, there is an alternative application: under certain stress conditions, their protein content decreases dramatically but their starch content increases by up to 50% of their dry weight or even higher [8,9]. Starch can be degraded to low-molecular-weight carbohydrates and thereafter fermented to bioalcohols, i.e., ethanol or even higher alcohols like butanol [10].

With the intention to cultivate duckweeds under optimal conditions for nutrition, i.e., with a high protein content, one has to deal with stress factors in the environment. To make large scale production of duckweeds possible, as required for a novel nutrition crop, farmers have to learn how to deal with these stressors. These include both abiotic factors, like high temperature, salinity, heavy metals, xenobiotics [11] and biotic factors [12] similar to terrestrial crop plants.

In the current era of global climate change, it is extremely important to foster strategies in line with the principles of sustainability in agriculture. One of the major fields is stress management from both abiotic and biotic stressors. The objective of this workshop was to bring together scientists from these fields and to discuss strategies of how terrestrial and aquatic plants can be integrated in a concept of agriculture for the future. In the following four sections—Abiotic stress management in terrestrial plants, beneficial interaction of fungi with plants, biotic stress management in terrestrial plants, and stress in duckweeds and other aquatic plants—we have reported the findings presented by different speakers at the workshop as indicated by their name and place in braces.

## 2. Abiotic Stress Management in Terrestrial Plants

Abiotic stresses reduce the crop yield significantly, partially because of the effects of global climate change. Rice (*Oryza sativa*) is an important staple food for over half the world’s population, and also for India. It has been discovered that several stress factors cause the accumulation of a cytotoxic metabolite, methylglyoxal. The concentration of this metabolite was reduced by engineering the glyoxalase pathway, and stress tolerance was enhanced in rice [13]. Additionally, it was demonstrated that Na^+^/H^+^ antiporters help in the sequestration of salt and enhance salt tolerance (Sneh Lata Singla-Pareek, New Delhi, India). In fact, almost two-and-a-half decades back whilst elaborating the role of Na^+^/H^+^ antiporters in the salt tolerance of halophytes, Glenn et al. [14] were already reporting on the potential application of halophytes, non-conventional crops, in the remediation of saline-struck soils and wastewater. With the present-day global challenges, this application could contribute to the development of sustainable strategies. Under environmental conditions, often several stress factors affect the plants, either in a combined or sequential manner. Besides drought and high temperature, it is expected that flooding, connected with the rivers Ganga and Brahmaputra, will create stress on crop plants. The use of modern methods of plant breeding like gene modification tools and mutation breeding is expected to provide a solution to manage stress in rice (Ashwani Pareek, Mohali, India; [15]). Drought stress responses in resistant and sensitive rice were investigated using high-throughput multi-omics (transcriptome, proteome, and metabolome) technologies. An integrated analysis revealed the importance of carbohydrate metabolism through glycolysis, the pentose phosphate pathway, and L-phenylalanine biosynthesis in the resistance response. Moreover, the dominant role of epigenetic regulations became evident (Mukesh Jain, New Delhi, India; [16]). Deepwater rice is naturally resistant to flooding and even seed germination under submerged conditions is possible. During the investigation of the involved mechanisms, it was discovered that the phytoglobin-NO cycle plays an important role. As a practical measure it could be shown that the addition of nitrite stimulates anaerobic germination and reduces ROS production, indicating the alleviation of stress (Kapuganti Jagadis Gupta, New Delhi, India; [17]). The cryopreservation of genetic resources is an ex situ conservation technique, commonly used for vegetatively propagated plants. The meristematic tissues that are used in the cryopreservation process have to survive through several stressors such as cold temperature, osmosis and wounding. Studies on the effect of these stressors on the success of the cryopreservation process are very crucial for the long-term storage of plants genetic resources. In order to develop successful protocols for the cryopreservation of plants, identifying the signalling cascades involved in cryopreservation could be significant. Whereas good progress has been obtained with *Arabidopsis* using shoot tips, progress is urgently awaited for duckweeds (Lemnaceae), which rarely flower, and the production of seeds is not yet established as a routine method (Manuela Nagel, Gatersleben, Germany). Brassinosteroids play an important role in the regulation of plant development and in their interaction with the environment. The dynamics of the brassinosteroid response pathway at the plasma membrane and the initiation of cellular responses related to cell elongation were identified using recurrently combined computational modelling together with quantitative cell physiology. The model shows the hyperpolarization of the plasma membrane that is induced by brassinosteroids and the swelling of the cell wall subsequent to the apoplast acidification (Klaus Harter, Tübingen, Germany; [18]). “Chlorophyll is a Janus-faced molecule of life and death”. Electrons from chlorophyll can be lethal to the cells, acting via the reactive oxygen species. Insect pests on plants have developed mechanisms to defend themselves from the phototoxicity of the ingested chlorophyll. It was found that two special proteins are involved in this process. Insects with knock-outs of these proteins could not survive in the presence of light after being fed on green leaves, unlike in the dark. This deepens the understanding of insect adaptation to herbivory (David G. Heckel, Jena, Germany; “Naked chlorophyll stresses insects as well as plants”). In intensive discussions, biological systems were proposed in which the relevance of these new findings could be tested for future agricultural applications.

## 3. Beneficial Interaction of Fungi with Plants

The association of endophytic microbes with crop plants is beneficial in building a sustainable agriculture system, owing to the ability of these microbes to quickly adapt to changing environmental conditions in comparison to their host plants. This association stimulates the physiological traits of the crop plants which results in significant improvement in the crop’s stress tolerance. Endophyte-enrichment technology can be a booster to plant productivity (Karaba N Nataraja, Bengaluru, India; [19]). Microbial interactions of the endophytic fungus *Piriformospora indica* with plants may alter or destroy the structure of the plant cell wall, connecting cell wall integrity maintenance to immune responses. Plant cell wall breakdown generates short-chain cellooligomers which induce Ca^2+^-dependent responses. These responses require the malectin domain-containing CELLOOLIGOMER-RECEPTOR KINASE 1 (CORK1] in *Arabidopsis*. Two conserved phenylalanine residues in the malectin domain are crucial for CORK1 function (Ralf Oelmüller, Jena, Germany; [20]). *Piriformospora indica* colonizes the roots of a broad host range and, additional to a higher biomass production, the symbiosis also confers resistance to abiotic and biotic stress conditions like high salinity or infection by pathogenic fungi. Furthermore, *P. indica* provides the host not only with water and phosphate, but also with other important nutrients like nitrogen. By use of an N-15-isotope-labelled fungus, it was demonstrated that the fungus directly transfers nitrogen from the hyphae to the host plant (Sandra Scholz, Jena, Germany). *Cyanodermella asteris,* is a fungal endophyte isolated from *Aster tataricus*, a plant known in Chinese traditional medicine that produces astin as an active molecule. The interaction of non-natural hosts *A. thaliana*, Chinese cabbage, rapeseed, tomato, maize, or sunflower with *C. asteris* results in phenotypes like a decrease in the main root length, extensive lateral root growth, increased leaf and root biomass, and enhanced anthocyanin levels—properties of high agricultural interest [21]. It was found that auxin secreted by *C. asteris* was involved in the phenotype of the root whereas signalling pathways for salicylic acid, jasmonic acid or ethylene were not found to be involved in the plant-microbe interaction (Jutta Ludwig-Müller, Dresden, Germany). Association of plants with Arbuscular mycorrhizal fungi help the plants with sourcing nutrients and also with increasing their resistance and tolerance to varied stresses. However, the plant–mycorrhizal interactions are not always positive. The current research is directed towards the performance of arbuscular mycorrhiza under the changing and challenging conditions of agricultural and horticultural plant production systems (Philipp Franken, Erfurt and Jena, Germany; [22]). Adverse effects of salinity on crops are mainly osmotic toxicities and the uptake of ions in concentrations that are not beneficial for the plants. Soil bacteria can help plants to better adapt to saline soils with high osmotic stress, poor physical conditions, nutritional disorders, and toxicities. A newly isolated and characterized Rhizobium from the common bean nodule from Kenya showed high symbiotic efficiency to relieve its host plant from acute salt stress (Clabe Wekesa, Jena, Germany; [23]). Non-brassinoid steroids were investigated in several terrestrial plants as well as in species of all five genera of duckweeds. It was shown that the infection of *A. thaliana* with *Alternaria brassicicola* (a phytopathogenic Ascomycota) specifically increases the amounts of dehydroepiandrosterone (an early androgen) in infected leaves, while the amounts of other steroids did not change. This steroid is a strong inhibitor of fungal growth (Jan Klein, Jena, Germany). This workshop session provided multiple strategies and novel chemical compounds relevant for agricultural applications. We discussed a common field experiment. The harvested material should be analysed by members of the workshop using their specialized knowledge.

## 4. Biotic Stress Management in Terrestrial Plants

Autotrophs are constantly challenged by abiotic as well as biotic stressors. Insect pests of plants are one of the major biotic stressors. Plants have evolved several strategies to protect themselves from insect infestation. However, the signal transduction pathways induced early on that connect the process of wounding as a result of insect herbivory to the plant defence responses are yet to be well comprehended. Perceived by specific chemicals (herbivore-associated molecular patterns or damage-associated molecular patterns), plants respond with local and systemic signalling processes including [Ca^2+^]_cyt_ changes and phytohormones like jasmonates. Moreover, volatile-mediated plant–plant communication play an important role in the biotic stress response (Axel Mithoefer, Jena, Germany; [24,25]). Plant- and microbe-based biologicals can boost crop productivity as well as mitigate the effect of stressors on plants. Prime Verdant (produced by *BioPrime AgriSolutions*, India) improved the productivity of tomato crops both qualitatively and quantitatively. Prime-Verdant-applied plants performed well also under stress conditions (Rahul Jog, Pune, India). Furthermore, vegetable crop plants can become heavily infected by different plant viruses which results in damage to the crops. It is difficult to mitigate RNA and DNA viral diseases in vegetable crops. *Piriformospora indica*-colonized tomato plants developed increased resistance to *Tomato yellow leaf curl virus,* and the colonization of fruit plants like banana and papaya with *P. indica* effectively reduced the disease symptoms induced by RNA viruses like *Banana bract mosaic virus* and *Papaya ring spot virus*, respectively. Additionally, vegetable crops (yard long beans, tomato and lady’s finger) in association with *P. indica* developed more resistance against RNA virus (*Cowpea aphid borne mosaic virus, Blackeye cowpea mosaic virus*), and DNA virus (*Tomato leaf curl virus* and *Bhindi yellow vein mosaic virus*) infections. Thus, *P. indica* colonization protects plants also against viral diseases (Joy Michal Johnson, Kollam, India). To understand the molecular basis of this *P. indica*-induced resistance, colonized and uncolonized crops after viral infections should be analysed with the molecular and biochemical tools available in the consortium.

## 5. Stress in Duckweeds and Other Aquatic Plants

Under suboptimal cultivation conditions, several duckweed species and clones accumulate starch, useful finally in biotechnology to produce bioalcohols via degradation and fermentation. Suboptimal cultivation conditions mean that the plants are under stress. Several stress factors are effective but in each case it is necessary to optimize their duration and strength for the different species and clones or ecotypes of duckweeds. The most obvious stress response in duckweeds is the inhibition of vegetative propagation (growth), i.e., by increasing salinity, a lack of nutrients or their exposure to heavy metals [26]. Evidently, in many cases, growth is more sensitive to stressors than photosynthesis is. As a consequence, not all photosynthetic products are required to support growth and the surplus is used to synthesize starch. The induction of starch-synthesizing enzymes like ADP-glucose pyrophosphorylase has been shown by transcriptomics and immunoblots. This inhibition of growth can be used also for the biomonitoring of toxic compounds in the aquatic environment as shown recently by *Lemna* root re-growth test (Klaus-J. Appenroth, Jena, Germany; [27]). The management of stressors can be executed by the integration of strategies like genetic engineering, e.g., of Na^+^/H^+^ antiporters in these unconventional crop plants that might increase their stress (salt stress)-tolerance capacity. The application of such management approaches is important, especially in the scenario where the ability of these plants to produce high amounts of biomass at a fast pace and the increasing practical applicability of this biomass in a circular economy are transforming duckweeds into a sustainable cropping system. The anatomical and molecular basis for the fast growth rate of these tiny aquatic plants was also revealed (K. Sowjanya Sree, Periye, India; [6,28,29]). With the intention to make duckweed biomass available on a large scale, an Indoor Vertical Farm was constructed, which produces duckweed biomass in a continuous manner and manages stress by nutrients and light (Finn Petersen, Osnabrück, Germany; [30]). Flowering in angiosperms is a robust process. Under stress conditions, when several of the vegetative parts of the plant show marks of stress on their morphology, flower development is intact. Each angiosperm species has a specific floral structure. The extensive diversity of flowers has evolved over “variation of the floral theme”. The ABCDE and Floral Quartet models were developed for model plants. However, these standard model themes have been modified during the evolution of some land plants, such as orchids, and also in some aquatic plants (Günter Theißen, Jena, Germany; [31]). In angiosperms, the evolution of aquatic plants had occurred several times independently during the transition of these plants from a terrestrial to an aquatic habit. The authors of this presentation made use of the enormous amount of available genome data for water plants, like different duckweed species, *Nelumbo nucifera*, and so on in order to annotate the MADS-box genes. The transcription factors encoded by these genes are involved in diverse plant developmental processes. The molecular bases for the morphological adaptations of water plants to the aquatic habit have been identified by the first ever genome-wide analysis of MADS-box genes in these aquatic plants (Lydia Gramzow, Jena, Germany; [31]). In a stand, the land plant communities show strong gradients of light intensity and light quality. This happens because of their competitive light absorption strategy. Plants under the stress of such light gradients, e.g., the exposure to sub-optimal light intensity that effects the plant’s photosynthetic efficiency, have evolved mechanisms to acclimatize and mitigate the diverse effects of these stressors. A light system that preferentially excites either photosystem I or photosystem II was established so as to study the acclimation responses of these stressed plants. This light system can induce short-term and long-term acclimation responses that include structural changes in the thylakoid membrane, changes in protein phosphorylation, photosystem II supercomplex formation, the structure of light-harvesting complex II and so on [32]. This understanding from the land plants, from the point of view of the knowledge and the experimentation abilities gathered has been systematically applied to carry out studies on aquatic plants like duckweeds. The effect of the aquatic habit and the two-dimensional canopy on the photosynthetic acclimation responses in duckweeds was investigated (Thomas Pfannschmidt, Hannover, Germany). Furthermore, the remarkable difference in the flowering behaviour of Lemnaceae and land plants offers various molecular approaches to investigate how investments into flower development or vegetative growth impact crop yield.

## 6. Conclusions

Knowledge flow between the researchers from both India and Germany on sustainable stress management in terrestrial and aquatic plants has helped to widen the understanding of the mechanism of stress management in general and also allowed inputs and learning into each field in order to work towards developing sustainable aquatic and terrestrial cropping systems that can deal with stress in a more efficient manner. The development of well-organized sustainable agriculture systems, which will enable us to create innovative technologies that can be integrated into circular economy, is the future. Our efforts to understand the stress-management strategies in both terrestrial plants and aquatic plants facilitated a holistic understanding of the requirements and strategies for building sustainable cropping systems.

## Data Availability

All the required data are presented in the manuscript.
